# Access to midwifery care for people of low socio-economic status: a qualitative descriptive study

**DOI:** 10.1186/s12884-019-2577-z

**Published:** 2019-11-12

**Authors:** Elizabeth K. Darling, Lindsay Grenier, Lisa Nussey, Beth Murray-Davis, Eileen K. Hutton, Meredith Vanstone

**Affiliations:** 1 McMaster Midwifery Research Centre, McMaster University, HSC 4H24, 1280 Main St W, Hamilton, ON L8S 4K1 Canada; 2 Department of Family Medicine, Centre for Health Economics and Policy Analysis McMaster FHS Education Research, Innovation & Theory (MERIT) program, McMaster University, 100 Main St. W, Hamilton, ON L8P 1H6 Canada

**Keywords:** Midwifery, Healthcare access, Low-socioeconomic status, Maternity care, Healthcare services

## Abstract

**Background:**

Despite public funding of midwifery care, people of low-socioeconomic status are less likely to access midwifery care in Ontario, Canada, but little is known about barriers that they experience in accessing midwifery care. The purpose of this study was to examine the barriers and facilitators to accessing midwifery care experienced by people of low-socioeconomic status.

**Methods:**

A qualitative descriptive study design was used. Semi-structured interviews were conducted with 30 pregnant and post-partum people of low-socioeconomic status in Hamilton, Ontario from January to May 2018. Transcribed interviews were coded using open coding techniques and thematically analyzed.

**Results:**

We interviewed 13 midwifery care recipients and 17 participants who had never received care from midwives. Four themes arose from the interviews: “I had no idea…”, “Babies are born in hospitals”, “Physicians as gateways into prenatal care”, and “Why change a good thing?”. Participants who had not experienced midwifery care had minimal knowledge of midwifery and often had misconceptions about midwives’ scope of practice and education. Prevailing beliefs about pregnancy and birth, particularly concerns about safety, drove participants to seek care from a physician. Physicians are the entry point into the health care system for many, yet few participants received information about midwifery care from physicians. Participants who had experienced midwifery care found it to be an appropriate match for the needs of people of low socioeconomic status. Word of mouth was a primary source of information about midwifery and the most common reason for people unfamiliar with midwifery to seek midwifery care.

**Conclusions:**

Access to midwifery care is constrained for people of low-socioeconomic status because lack of awareness about midwifery limits the approachability of these services, and because information about midwifery care is often not provided by physicians when pregnant people first contact the health care system. For people of low-socioeconomic status, inequitable access to midwifery care may be exacerbated by lack of knowledge about midwifery within social networks and a tendency to move passively through the health care system which traditionally favours physician care. Targeted efforts to address this issue are necessary to reduce disparities in access to midwifery care.

## Background

Low socioeconomic status (SES) is a fundamental cause [1] of adverse birth outcomes, including gestational diabetes, small for gestational age, low birth weight, intrauterine growth restriction, preterm birth, asphyxia, and neonatal mortality [2–5]. The causal pathways between low SES and perinatal outcomes are varied and complex [6–8]. Appropriate prenatal care can improve outcomes [9, 10]; however, even within a publicly funded health care system, people of low SES are less likely to receive adequate prenatal care due to a range of structural, economic, psychosocial, and attitudinal barriers [11]. Recent evidence shows that continuity of care midwifery is associated with lower risks of small-for-gestational age, preterm birth, and low birthweight for people of low SES [12, 13]. The characteristics of midwifery care, including longer appointments and a non-judgmental approach, may help to overcome barriers that prevent women of low-income from accessing adequate prenatal care [14]. Informed choice, choice of birthplace, continuity of care, and flexible community-based care that includes home visits are additional characteristics of midwifery care that enhance the ability of midwives to meet the needs of marginalized people [15, 16].

Midwifery services in Canada are regulated and funded at the province/territorial level [17]. Midwives in the province of Ontario are self-regulated by a College of Midwives and midwifery education involves a four-year baccalaureate degree or equivalent [18]. The standard model of midwifery care in Ontario involves continuity of care, in which a team of up to four midwives is fully responsible for a woman’s care throughout pregnancy, labour, birth, and the first six weeks following birth [18]. Midwives work in community-based midwifery practice groups and all offer intrapartum care in both hospitals and community-based settings (home, birth centre, or clinic) to women with low-risk pregnancies [19]. The cost of midwifery care is publicly funded for all residents of Ontario [20]. Conditional upon availability, individuals are free to choose between care from an obstetrician, a family physician, or a midwife for their pregnancy and birth [20]; however, only midwives provide intrapartum care outside of hospitals. Only a minority of family physicians provide intrapartum care. In 2017, midwives attended 16% of births in Ontario and 10.8% nationwide [17], and 83% of midwife-attended births in Ontario occurred in hospital [21]. Low midwifery care attendance across all populations is impacted by the relatively recent integration of midwifery into the health care system in Ontario in 1994 [22]. Previous research, not specific to low SES, identified barriers to women’s acceptability and usage of midwifery care, including poor levels of awareness and knowledge, including misconceptions [14, 23–26] and concerns about the safety and expertise of midwives in emergencies [23–25, 27, 28].

Access to midwifery care in Ontario based on SES has not been well described. In a 1999 survey conducted five years after the regulation and formal integration of midwifery into the Ontario health care system, midwives reported that public funding had increased the diversity of the midwifery client population, with 94% of midwifery practice groups reporting increased utilization by low income women [15]. Although increasing access to midwifery care for women from disadvantaged groups was explicitly identified as a goal of regulation of midwifery [29], unpublished analyses by our research team suggests that people of low SES in Ontario are less likely to access midwifery care. Little is known about barriers pregnant people of low SES might experience in entering into publicly funded midwifery care and how they can be overcome. The goal of this study was to understand the barriers and facilitators to accessing midwifery care experienced by people of low SES. Our primary research question was “What are the barriers and facilitators identified by pregnant people of low SES related to accessing midwifery care?”

## Methods

We conducted a qualitative descriptive study using semi-structured interviews [30, 31]. Ethics approval for the study and all related documents was obtained from the Hamilton Integrated Research Ethics Board. In acknowledgement that not all people who become pregnant identify as women, we refer to participants as ‘people’ or ‘pregnant people’, rather than ‘women’ throughout this paper. The gender identity of our participants was not collected.

### Participants, setting, recruitment

People were eligible to participate in the study if they were currently pregnant or had given birth within the past year and were of low SES, regardless of their choice of health care provider. We recruited residents of Hamilton, Canada, a southern Ontario city with a population of 535,000 from January to May 2018. We recruited through social media and posters at health care and social services sites including midwifery clinics, an inter-professional community-based maternity care clinic, hospital-based obstetrical clinics and birth units, community organizations, and prenatal programming. Participants were offered a small, $25 cash honorarium in recognition of their time, in addition to any transportation costs incurred in participating in the interview. Potential participants directly contacted the study team through social media, email, and telephone. We screened people upon initial contact by using five questions related to SES to determine their eligibility to participate.

SES is a multifaceted construct used to define social inequality and is measured by income, occupation and educational attainment [6, 32]. Screening questions addressed highest education level achieved, employment status, occupation, receipt of government income support, and household income [33]. People who were below the low income cut-off for the city of Hamilton [34] or received government income support [33], and whose highest level of education was high school or below were eligible to participate in the study [35]. Low income cut-offs from Statistics Canada were used to determine low-income status for the city of Hamilton, based on household income and family size living in the house [33, 34, 36]. We were interested in recruiting participants whose highest level of education was high school or below, but due to the complex way in which SES is measured, and given the high rate of high school completion in Canada (88% in 2010) [37] we also included some individuals who did not meet this criterion. For people who had low income or received government income support but had completed higher levels of education, the principal investigator reviewed responses related to occupation and employment status, and participants were enrolled if they were unemployed, employed part-time, or employed in industries with low-wages or precarious work [33, 38, 39].

### Data collection

Participants received the consent form and all study information in advance of the interview to review. Informed consent was obtained from all participants prior to the interview, either written or verbally depending on whether their interview was in person or over the phone. Verbal consent was audio recorded and obtained by the research assistant who read and explained the consent form with the subject, who gave their verbal consent to participate. We had ethics committee approval to obtain consent in this manner. Participants were informed they could end the interview or withdraw from the study at any time, even after consent was obtained. All interviews were audio recorded. An experienced research assistant conducted individual interviews using a semi-structured interview guide. We made a methodological decision that the interviewer would not be a midwife, or any other type of healthcare practitioner, to increase participant comfort in expressing candid thoughts and opinions about midwifery care. We offered the choice of an in-person or a telephone interview. The semi-structured interview guides explored participants’ barriers and facilitators to accessing care with their chosen HCP, their reasons for choosing their HCP, and their experiences of care. Several questions were specific to people who had received care from other clinicians, such as family physician, obstetrician, or nurse practitioner. These questions explored whether they had ever considered using a midwife, information they received from health care providers about their options for pregnancy and birth care, and their knowledge and opinions of midwifery care. Recruitment continued until data saturation in each of the care provider groups was achieved [40]. Data saturation was established through constant iterative review of the interviews to ensure no new data was emerging [41–43].

### Analysis

Interviews were professionally transcribed. Transcripts were analyzed and managed using NVivo 12 software. The data were analyzed using qualitative descriptive content analysis, beginning with open coding to summarize and describe the data, and then proceeding with focused coding to identify and categorize themes [30, 31, 44]. The research assistant read each transcript in full and coded the data, and met regularly with the principal and co-principal investigators throughout the process to develop and refine the coding scheme. We compared findings within, and then between the different care provider types. Descriptive statistics were used to summarize the sociodemographic characteristics of the participants.

### Criteria for rigor

We used Whittemore and colleagues’ criteria for conducting high quality qualitative research to achieve rigor in this study: credibility, authenticity, criticality and integrity [45]. These criteria are commonly paired with qualitative description [46]. Credibility and authenticity are closely linked; we ensured that we remained true to the purpose of the research by ensuring the rich data that reflected the perspectives was collected and accurately represented throughout analysis and reporting. We did this by encouraging participants to tell their own stories, emphasizing their own priorities rather than responding to researcher ideas about which aspects of the experience of accessing prenatal care are important. We made sure that the multivocality of participant voices is present in our findings by paying particular attention to our own influence as interviewers and analysts throughout the study. We chose to use a non-clinician interviewer and a multi-disciplinary team of analysts to ensure that no single perspective overrode the voice of participants. Criticality and integrity were achieved through the use of an iterative research design which allowed us to search for discrepant opinions, conflicting interpretations, and perform recursive and repetitive checks of our interpretations as they evolved.

## Results

One-hundred and forty-nine people were screened for potential eligibility. Of those, 23 did not respond to the screening questions, and 84 were ineligible. Eight were eligible and invited to participate in an interview but were lost to follow-up and an additional 4 were eligible, but not interviewed due to reaching saturation. Thirty people participated: 13 had received care from midwives and 17 had never received care from midwives. Among those who had never seen midwives, 10 (33%) had seen obstetricians and 7 (23%) had seen a family physician. Eight participants had experienced both midwifery and physician care, either in different pregnancies or as a consequence of a transfer of care. Several participants considered midwifery care or attempted to enter midwifery care but were rejected due to an assessment by either a midwife or physician, that deemed them high risk and not suitable for midwifery care. Interviews ranged from 11 to 76 min, with a mean length of 26 min.

The sociodemographic characteristics of participants are shown in Table 1. The mean age was 29 years with a range of 17–46, and the majority were unemployed (80%), had a combined household income of less than $30,000 CND (60%), and rented their homes (73%). Twenty-seven percent were born outside of Canada. Seventy-three percent of participants lived in neighbourhoods that were in the bottom two quintiles of overall rankings based on 24 health, social, and economic variables [47].

**Table 1:** Characteristics of Study Population (*n* = 30)

Characteristic			
	Midwife care provider (*n* = 13) n (%)	Physician care provider (*n* = 17) n (%)	Total (n = 30) n (%)
Age in years			
< 20	2 (15.4)	2 (11.8)	4 (13.3)
20–30	5 (38.5)	8 (47.0)	13 (43.3)
30>	6 (46.1)	7 (41.2)	13 (43.3)
Annual Household Income			
Unknown	0 (0)	1 (5.9)	1 (3.3)
< $19,999	5 (38.5)	10 (58.8)	15 (50.0)
$20,000–$29,99	0 (0)	3 (17.6)	3 (10.0)
$30,000–$39,999	4 (30.8)	1 (5.9)	5 (16.7)
$40,000–$49,999	1 (7.7)	2 (11.8)	3 (10.0)
≥ $50,000	3 (23.1)	0 (0)	3 (10.0)
Country of Birth			
Canada	10 (76.9)	12 (70.6)	22 (73.3)
Other	3 (23.1)	5 (29.4)	8 (27.7)
Residency Status of non-Canadian-born			
Canadian Citizen	2 (15.4)	2 (11.8)	4 (13.3)
Work Permit	0 (0)	1 (5.9)	1 (3.3)
Student Visa	0 (0)	1 (5.9)	1 (3.3)
Refugee Claimant	0 (0)	1 (5.9)	1 (3.3)
Permanent Resident	1 (7.7)	0 (0)	1 (3.3)
Language Spoken at Home			
English	11 (84.6)	12 (70.6)	23 (76.7)
Other	2 (15.4)	5 (29.4)	7 (23.3)
Highest Level of Education			
No high school	1 (7.7)	0 (0)	1 (3.3)
Some high school or high school diploma	7 (53.8)	3 (17.6)	10 (33.3)
Some college or college degree	2 (15.5)	7 (41.2)	9 (30.0)
Some university or university degree	3 (23.1)	4 (23.5)	7 (23.3)
Some graduate school or graduate degree	0 (0)	3 (17.6)	3 (10.0)
Parity			
Nulliparous	5 (38.5)	12 (70.6)	17 (56.7)
Multiparous	8 (61.5)	5 (29.4)	13 (43.3)
Employment			
Employed	4 (30.8)	2 (11.8)	6 (20.0)
Unemployed	9 (69.2)	15 (88.2)	24 (80.0)
Parental Leave	3 (23.7)	5 (29.4)	8 (26.7)
Home Ownership			
Rent	7 (53.8)	15 (88.2)	22 (73.3)
Own	4 (30.8)	2 (11.8)	6 (20.0)
Other	2 (15.4)	0 (0)	2 (6.7)
Living Situation			
People living with partner	8 (61.5)	10 (58.8)	18 (60.0)
People not living with partner	5 (38.5)	7 (41.2)	12 (40.0)
People living in extended family situations^a^	3 (23.1)	4 (23.5)	7 (23.3)
Food Insecure within previous 12 months			
Yes	4 (30.8)	4 (23.5)	8 (26.7)
No	9 (69.2)	13 (76.5)	22 (73.3)
Receives Income Support from Government			
Yes	5 (38.5)	6 (35.3)	11 (36.7)
No	8 (61.5)	11 (64.7)	19 (63.3)

^a^a single household that may include family, friends, and partner

Note: Totals may not add up to 100% due to rounding

We identified four major themes arising from the interviews: “I had no idea…”, “Babies are born in hospitals”, “Physicians as gateways into prenatal care”, and “Why change a good thing?”. Each theme is discussed in greater detail below using illustrative quotations from the interviews. The source for each quote is identified with ‘MW’ or ‘MD’ to denote ‘midwifery client’ and ‘physician patient’, respectively.

### “I had no idea…”

One of the strongest themes influencing access to prenatal care providers centred around a lack of knowledge about midwifery. We repeatedly heard that participants and members of their social network had no idea what midwifery care entails. Lack of knowledge was also evident in frequently expressed misconceptions about midwifery care. The most influential mitigating factor to lack of knowledge about midwifery when choosing a prenatal care provider was personal referral from a friend or relative.

The majority of participants (87%) had heard of midwives, but their understanding of midwifery varied greatly. While some participants who had never received midwifery care understood the role, their overall knowledge levels about midwifery were low. Of participants who received care from a physician, 70.6% had little to no knowledge of midwifery. Some participants did not know what midwives do or provided an inaccurate description of midwifery services. Common misconceptions were that midwives only attended home births; that they only provide ‘natural’, drug free labour and delivery; that one must pay privately for midwifery care; that a doctor’s referral is required to see a midwife; and that midwifery is unsafe. Knowledge about midwifery among newcomers to Canada was influenced by the role of midwives and respect for the profession in their country of origin. Misconceptions regarding the Ontario system included that midwives were assistants to obstetricians, that they were nurses, that they were not certified, and that they were the option used by poor people who couldn’t afford better care. As one participant said:

*Midwifery care in many South American countries is still perceive as a “lesser” profession compared with an OB, so most women under the same circumstances as mine, choose OB care. (MW6*)

The skills and competencies of midwives were poorly understood. While most participants had a general sense that midwives were educated and “certified,” they were not sure what this meant. In comparison with physicians, many participants felt uncertain about how to evaluate their trust in the qualification of midwives:

*But it’s a good question to ask, right? What’s the education like? … How long have you been doing it? Are you allowed to ask for proof? Can I see your license? Like it just feels weird, and I feel with an OB you don’t have to do any of that, that all comes with it. (MD28)*

Those who were aware of midwifery reported learning about the profession from a variety of sources, including books, websites, tv shows, social media, prenatal classes, brochures, and family physicians; however, word of mouth from family and friends was the most common and most influential source information. While misinformation discouraged some participants from pursuing midwifery care, hearing about the experiences of others who had actually experienced midwifery care and receiving a positive personal recommendation increased people’s comfort with the services midwives provide:

*[W]ithout her experience, I would have thought it was too dangerous… having someone describe their experiences and actually fill me in on all my questions of how it happened and what they did…that was huge. So yeah, I don’t know if I actually would have researched it as much if someone close to me hadn’t had talked about it. (MW10)*

People often encountered skepticism and judgment from their friends and family members about choosing midwifery care. For those who felt strongly about midwifery, offering information to their skeptical partner or family was an important part of getting commitment:

*…once they met my midwives and they saw my first birth, it was like why wouldn’t you have your baby at home? You know, that this is the way to do it. So it totally changed their perspective and their view on midwifery and the care. (MW02)*

Participants also received information about midwifery from social service agencies, such as the Children’s Aid Society or government-sponsored services targeting young parents. Knowledge and awareness of midwifery in our participants involved with these agencies was much higher than in the rest of our study group. All four of these participants were given options and information about all care providers, resulting in two choosing midwifery, one being unable to find a midwife despite a preference for this care provider, and one choosing an obstetrician. However, several participants noted that hearing about midwifery for the first time at the point when choosing a care provider meant they were less likely to feel comfortable with the unknown option. As one participant said:

*I don’t know about any other provider, like midwife. This is the first time that I heard that something like that exists…. I just wanted to have a doctor at first because like I said, I wasn’t familiar with midwives and what they do. (MW01)*

### “Babies are supposed to be born in a hospital”

Another theme arising from the interviews pointed to the influence that prevailing attitudes and beliefs about pregnancy and childbirth had on people’s choices and behaviour related to accessing maternity care. These ideas encompass both the norms and expectations in Canadian society regarding what kind of health care is ideal, and the underlying beliefs about risk and safety.

When discussing decision-making regarding their choice of care provider, many participants expressed the notion that going to the doctor and planning to give birth in hospital was a normal and expected part of pregnancy and childbirth. For many of our participants this was the only pathway they were aware of when they became pregnant. Several participants described following this path as ‘easy’:

*So it’s like total path of least resistance. I got my OB. He’s at a hospital already. It’s 15 min away from me, that’s that. I didn’t look into it any further. (MD28)*

Concerns about risk and safety were frequently raised as participants explained their choice of care provider. One particular area of concern for participants was homebirth. Midwifery care is often conflated with homebirth, despite the fact that all midwives in Ontario provide intrapartum care in hospitals. Homebirth was not the only safety concern, with participants also reporting that emergencies, complications, and the chance of being high risk were reasons for choosing an obstetrician over a midwife:

*I mean I’m all for midwives if you know that it’s going to be pretty easy sailing. But I just felt more comfortable with a doctor… I was concerned about being high risk. Again, that’s why I didn’t choose the midwife. (MD12)*

Only some participants understood that midwives in Ontario are only responsible for low-risk pregnancies, and that care would be transferred to a physician if needed. Participants who had no experience of midwifery care expressed doubt regarding midwives’ ability to assess risk level or judgement of when to transfer care to an OB:

*But I just have a problem with judgement of a midwife if you are high risk. If you’re not high risk, it’s her judgement to see if you should be referred to OB or not. (MD09)*

Participants expressed more frequent concerns about safety for first-time mothers, with many believing that midwifery wasn’t a good option for a first pregnancy:

*[T]he reason...I want to go to hospital is just because it’s my first baby. I’m like completely new, so it’s just you feel a little bit more secure in case anything...I think for this delivery, because it’s my first, it makes me feel a little more secure to have it in the hospital. (MD22)*

### Family physicians as gateways into prenatal care

A third theme arising from the interviews was that family physicians are the gateway to the healthcare system and channel access to other health care providers. In Ontario, family physicians are frequently the primary point of contact with the health care system and access to specialists requires a referral from a family physician. Lack of clarity regarding referral processes, and family physicians’ knowledge and attitudes regarding midwifery care were identified by participants as impacting their access to midwifery care.

For participants with a family doctor, the advice of their family physician often impacted their choice of care provider. As one participant observed:

*...a lot of people, their family physicians would refer them to an OB, so they just go with what their family physician refers them to. (MW26)*

Many assumed that their family physician would explain all the maternity care options they thought were appropriate for them, and since few family doctors brought up midwifery, participants often never looked beyond their doctor’s initial recommendation. Participants also reported family physicians playing a “triaging” role by informing them that they were not suitable candidates for midwifery due to their high-risk status. The explanations provided for this high-risk status did not always match midwifery scope of practice or intake criteria.

Participants without a family physician initially sought care in pregnancy at a walk-in clinic or a hospital emergency department. These individuals did not receive any information or counselling on midwifery care, and were directed to find a family doctor to look after them:

*I actually didn’t have a family doctor at the time. I went to a walk-in clinic for confirmation of my pregnancy, but they didn’t give me that option [midwifery]. They just said get a family doctor and go through your pregnancy that route. (MD08)*

Although referral from a physician is not required to access midwifery care in Ontario, several participants were unaware of this.

Another major misunderstanding that emerged was the idea that people couldn’t have a family doctor *and* a midwife simultaneously. While it’s true that either a doctor *or* a midwife will provide prenatal care, women don’t cease being a patient of their family physician while getting care from a midwife. Several participants, particularly newcomers to Canada and those without family physicians interpreted this as “you can’t have a family doctor if you have a midwife”:

*I made the call to [a midwifery clinic]... and they said no because you already have a family physician. That’s what they told me. I said okay, I didn’t know that. I thought it’s available to anyone that wants one. And they said no, it has to be either or. (MD18)*

Some participants reported that their family physician did not respond positively when they informed them of their choice to pursue midwifery care. As one participant described:

*She was like “Oh?” And I was like “Yeah.” And then she just kind of looked slightly offended. She didn’t really say anything. She was just surprised, I guess. She didn’t say anything. She just said “Oh.” (MW27)*

Only 10 of 23 participants who had family physicians were informed of the option of midwifery care by their physician. Fewer received detailed information about midwives and the services they provide. As one participant explained:

*They didn’t really say that it was an option. They just said if you want to go with a midwife, do it now because they take care of you, we wouldn’t. So they didn’t explain how to get a midwife or any benefits or anything like that. It was just kind of like are you coming with us or are you going somewhere else? (MW07)*

Participants with greater levels of awareness of midwifery shared a common understanding that they had to call a midwifery clinic immediately upon learning of their pregnancy:

*I called right away. I called at like five weeks, as soon as I peed on the stick, which is what my friends told me to do. They were like “Call as soon as you know.” (MW03)*

The demand for midwives in Canada is greater than the supply, and midwifery care is often not available if people seek it later in their pregnancy, limiting the access of those who are not informed about midwifery when they first contact the health care system. Therefore, not being informed about midwifery care by family physicians in early pregnancy can restrict access to midwifery.

### **“**Why change a good thing?”

Our final theme, “Why change a good thing?” speaks to the role that satisfaction and dissatisfaction with previous healthcare experiences play in how people access health care in pregnancy. Features of the services provided shaped participants’ opinions about different types of health care providers. This theme relates primarily to participants’ perceptions regarding the appropriateness of care. It speaks to the fit between services and client needs and reflects the notion that access to care encompasses the ability to choose acceptable and effective services [48]. This theme was the one topic for which participants spontaneously offered opinions regarding the relationship between SES and access to care and midwifery.

Participants’ previous personal health care experiences had a large impact on their choice of care provider, across all care providers. Negative or unsatisfying experiences made participants more likely to look into other options or ultimately switch to a different care provider:

*[A]fter my disappointment with my first OB, I kept it in my mind that if I ever got pregnant again, I wasn’t going to go to an OB, I was going to go to a midwife because I wanted the one-on-one care. I wanted the personal care. (MD24)*

Alternately, if people had a positive experience, they were more likely to stay with the care provider they had previously. As one participant explained:

*I looked into the midwifery 15 years ago with my first daughter and I had a good experience, so for the rest of my pregnancies I just continued to use a midwife instead of an OB. (MW26)*

Midwifery care was appealing due to the services, values, and beliefs of the profession. Participants had positive opinions of: 24-h access to telephone support for urgent concerns; choice of hospital or home birth; continuity of care throughout pregnancy, birth and postpartum; comprehensive postpartum care including home visits and breastfeeding support; and the philosophy of informed choice. Participants stressed that these features were important drivers for seeking midwifery care. The degree of support and comprehensiveness of care were highlighted as services that went above and beyond what was typically expected from physicians:

*...it feels more involved in the sense that it doesn’t feel like one facet of care. It doesn’t feel like it’s just about the birth and there’s nothing else...They ask me how I’m doing mentally. They make sure I’m informed about each option along the way. When I bring my questions they’re always very informed to let me know if there are seminars...to get more information about whatever I’m asking about. (MW04)*

Participants liked the combination of broad clinical knowledge and emotional care-giving:

*I like that they were both mom and baby focused, that they knew all the science. They knew everything about what you need... scientifically and medically, but like it wasn’t just completely shut off when it came to your own feelings and concerns and questions and everything. It was more about a relationship rather than just a doctor’s visit. (MW08)*

Several participants noted that the comprehensive approach to care provided by midwives involved connecting clients with additional health and social services. This was seen as a benefit for people of lower income:

*I think lower income people would totally benefit from having midwifery care …I know midwives can offer a lot of resources to their clients, resources that I had no idea about, maybe resources that lower income families don’t know about as well. I feel like everybody in general needs support, but I think sometimes lower income families need a little bit extra support. (MW02)*

Participants who had experienced midwifery care stressed that the personal relationship they developed with their midwife was a large reason for entering midwifery care:

*The most important thing for me was having a relationship with the person that was going to be with me when I give birth... what drove me to midwifery care was that I can get to know my midwife and she gets to know me and my family and what we expect. (MW06)*

Positive relationships and continuity of care gave women a sense of confidence and trust that they would know the person delivering their baby. Participants described their interactions with midwives as comfortable, less clinical, hands-on, caring, respectful, and informative. Satisfaction with physician-led care varied, and some participants were very satisfied with their physician. Physician led care was most commonly described by participants as being hands-off, more clinical, and impersonal. Common complaints were that physicians didn’t take participants’ concerns seriously, and they did not provide options. Physicians were described by several participants as being poor communicators, which left them confused and not as informed as they would like:

*I wish that they took the time to explain things and not just rush you in and rush you out and just kind of blow you off... I had a lot of concerns because I wasn’t getting proper communication. I wasn’t getting proper care and my doctor just kind of blew it off and didn’t really take my concerns to heart or anything, didn’t listen to me. (MD19)*

Bound up in these concerns was a common complaint for recipients of physician care regarding appointment length and time waiting for appointments to start. Long wait times and short appointments negatively impacted patient satisfaction. Participants reported feeling rushed during appointments, had difficulty getting their questions answered and were dissatisfied that students or nurses would often be the ones providing answers rather than the physician. As one participant described:

*And I had to wait two and a half hours, three hours sometimes to see [my obstetrician]... I think the last time I actually saw her, I only saw her for five minutes and then the nurse was in actually doing my initial exam. Like pretty much every time I went I’d only actually see her for five minutes and she wouldn’t let me ask questions. (MW15)*

In contrast, participants reported that midwifery appointments were longer with short wait times, allowing them to develop a relationship with their midwives and time to ask questions.

Across all care provider types, participants preferred pregnancy and birth service locations that were closer to their homes. However, those who chose midwives were willing to travel to whichever clinic accepted them:

*We called two groups, the closest to where we live. We wanted to have the closest and they had a waitlist, so we just needed to go for the one that is the farthest away and they accepted me the same day when we applied. (MW01)*

This often resulted in them having to travel longer distances, but the general sentiment was that it was worth this effort to have access to care that was satisfying to them. The only notable accessibility issue concerning the location of the practice and transportation was the cost of parking. This issue primarily impacted obstetric clinics, but was also an issue for some midwifery clinics. The cost of parking was exacerbated by long pre-appointment wait times for obstetrical patients.

## Discussion

Our research explored factors that impacted access to midwifery care for people of low SES. Lack of awareness or knowledge, misconceptions, and personal beliefs about risk and safety were the predominant barriers to midwifery care identified by participants. Our findings are consistent with previous research regarding knowledge levels and misconceptions regarding midwifery, in addition to concerns about safety and level of expertise [14, 23–25, 27, 28]. Our findings are also in line with existing literature which have found that choice of care provider was associated with birth-related beliefs and expectations [28, 49]. While our findings are particular to the Canadian context, they may also be of relevance in other settings where midwives do not provide the majority of care within the maternity care system or where midwifery-led continuity of care models have been introduced recently.

While the barriers identified by our participants may be seen to be reflective of the barriers to midwifery care that are common to people of all SES levels, on further examination, our interviews revealed several reasons why people of lower SES may have poorer access to midwifery care in our context. One key reason is that people of low SES may be less likely to encounter people within their social networks who have experienced midwifery care within the Canadian context. Participants under the care of midwives identified learning about positive experiences of midwifery care via word of mouth as the most influential factor in convincing them of the benefits of midwifery care and dispelling their misconceptions about its safety. Previous research has also shown second-hand experience to be associated with more positive opinions about midwifery [25]. Lack of contact with trusted sources who have experienced midwifery care therefore reduces the chances that the common barrier of misinformation about midwifery care will be mitigated for people of lower SES.

Discussions about the expected norms for pregnancy care highlighted that many participants moved passively through the health care system along the path of least resistance. Participants who had accessed midwifery care stood in contrast to this, with 86% having sought out midwifery care on their own prior to any consultation with their doctor. Previous research has shown that patients often defer responsibility for decision making to their doctor and take a passive role in their health care [50]. This is particularly true for individuals of lower SES, who are more likely to have low levels of health literacy [51–54], lower levels of engagement [55], greater trust in physician expertise [50, 56], and less involvement in decision-making [55]. Our study does not allow us to make any broad conclusions regarding family physicians’ knowledge and attitudes towards midwifery care; however, previous research has found a lack of understanding and trust in the education and scope of practice of midwives [57–59]. With 57% of participants with family physicians in our study reporting that their family physician didn’t even raise the option of midwifery, our findings suggest that some family physicians are uninclined to refer patients to midwives. Given lower levels of empowerment with respect to health care, infrequent endorsement of midwifery care by family physicians may differentially impact people of low SES.

Limited availability of midwifery care may also have a greater impact on access to care for people of low SES. Sixteen percent of all births in Ontario are now attended by a midwife [22], but demand for midwifery care has continually outpaced supply [58, 60]. People of low SES are more likely to have an unplanned pregnancy [61–63], which may result in delays in seeking care. In addition, lower levels of general awareness of midwifery prior to pregnancy may also contribute to people of low SES seeking midwifery services later in pregnancy, at a timepoint when midwifery practice groups have no room left in their caseload to accommodate additional clients.

In spite of these barriers, participants who had experienced midwifery services found them to be very appropriate for people of low SES. A broad conceptualization of access to care includes the notion that once services are reached, they should effectively meet the needs of the service user [48], and our findings suggest that there is a good fit between the characteristics of midwifery care and the needs of people of low SES. For example, participants identified that midwives comprehensively coordinate care which facilitates access to additional resources for people of low SES. Timeliness of appointments, and satisfaction with interpersonal relationships were also elements of midwifery that were viewed favourably. Previous research has identified long waits prior to prenatal appointments as a barrier to prenatal care for marginalized women [64, 65], while shorter wait times, and longer appointments were among the top reasons for entering midwifery care [66]. Research also suggests that dissatisfaction with care and poor relationships with health care providers are barriers to prenatal care for people of low SES [65]. Our findings align with previous work showing that continuity of care facilitates access to care for people of low SES, as it provides consistency and allows patients to build connection and trust [64]. Other key features of midwifery care that have previously been identified as facilitators of access to care for people of low SES include sharing information, educating patients, connection with the care provider, care provider support, and lack of judgement [27, 64]. Overall, the appropriateness of midwifery care drives one of the key facilitators of access to care that we identified: word of mouth.

Our study has several limitations. One limitation relates to how we applied our inclusion criteria to screen for low-SES. We included some participants who had higher education but would otherwise be considered low-SES. This may have limited our ability to identify barriers experienced by people with lower levels of education, particularly given that education levels were slightly higher for participants who had not received midwifery care. Another limitation was that all participants had to be fluent in English, as we were unable to provide translation services, so our findings likely do not fully reflect the experiences of newcomer populations with lower levels of English fluency. Another limitation is that we did not apply an intersectional analysis to examine the impact of other social determinants of health, particularly racialization, in our study [67]. This is because we limited the scope of our research based on our available resources, which constrained our ability to recruit and interview enough participants to compare how access to care was impacted by racial identity. It should be noted that there is racial inequality to access to health care in Ontario [67], and while there have been some positive initiatives within the profession of midwifery in Ontario to support care that meets the needs of a diverse population (e.g., the undergraduate midwifery curriculum emphasizes cross-cultural competence, and there is a bridging program that supports foreign-trained midwives to enter the profession), the profession has been critiqued for creating a model of care that is based upon the needs of white, educated women and for under-representation of racialized groups within the profession itself [68]. Ontario has a highly diverse population [69], so this is a rich and important area of exploration for future research. We anticipate that research that specifically involves racialized people of low-SES could reveal additional barriers to midwifery care.

Our findings have several implications for policy, practice, and research. To date, the unmet demand for midwifery services in Ontario has limited the need for efforts to raise awareness about midwifery. Our findings suggest that in the absence of targeted efforts to improve awareness about midwifery care, people of low SES will continue to have inequitable access to these services. Midwives and midwifery professional associations should engage in knowledge translation and educational activities to improve the public’s knowledge and understanding of midwifery, which are designed to target people of low SES. Such efforts should prioritize dispelling myths that reduce the perceived acceptability of midwifery care. Personal stories of positive experiences may be a good format to convey information. Our findings also highlight the key role that family physicians play as the gateway to the healthcare system. Further qualitative research to explore the reasons why some family physicians do not currently refer patients to midwives would be helpful and could inform a response to this issue. Targeted knowledge translation activities aimed at family physicians should also be implemented to improve to access to midwifery care for people of low SES. Such activities should be designed to increase family physician’s knowledge regarding midwifery (including the potential benefits for people of low SES) and their confidence discussing pregnancy and birth care provider options with their patients. These activities should be based on existing evidence regarding effective knowledge translation. Midwives should also continue efforts at the community level to facilitate referral pathways to midwifery care for people of low SES by building networks with other service providers. Research to evaluate the impact of knowledge translation activities targeting family physicians would be helpful to inform the scale up of such activities.

## Conclusion

Our research identified a number of barriers and facilitators that impact the accessibility of midwifery care to people of low-SES. We found that when people of low SES experience midwifery care they find these services to be acceptable and appropriate. However, in our context, access to midwifery care is constrained for people of low SES because lack of public awareness about midwifery limits the approachability of these services, and because information about midwifery care is often not provided by physicians when pregnant people first contact the health care system. Inequitable access to midwifery care for people of low SES is exacerbated by lack of knowledge about midwifery within social networks and a tendency to move passively through the health care system which traditionally favours physician care. Targeted efforts to raise knowledge levels about midwifery among people of low SES and to change physician referral behaviour will be necessary to reduce disparities in access to midwifery care.

## Data Availability

The datasets generated and/or analysed during the current study are not publicly available due lack of consent from the study participants to share the data publicly but are available from the corresponding author on reasonable request.

## Abbreviations

SES: Socioeconomic status
